# Distribution of *Aedes albopictus* (Diptera, Culicidae) in southwestern Pacific countries, with a first report from the Kingdom of Tonga

**DOI:** 10.1186/1756-3305-5-247

**Published:** 2012-11-06

**Authors:** Laurent Guillaumot, Reynold Ofanoa, Lucien Swillen, Narendra Singh, Hervé C Bossin, Francis Schaffner

**Affiliations:** 1 Institut Pasteur de Nouvelle Calédonie, 9–11 Avenue Paul Doumer, Association Pasteur International Network ,Nouvelle-caledonie, Noumea, BP 61, 98845, New Caledonia; 2 Ministry of Health, Nuku’alofa, Kingdom of Tonga; 3 Retired WHO Technical Officer, Tontouta, New Caledonia; 4 Secretariat of the Pacific Community, Suva, Fiji; 5 Institut Louis Malardé, Papeete, Tahiti, French Polynesia; 6 Institute of Parasitology, University of Zurich, Zurich, Switzerland

**Keywords:** Aedes albopictus, Vector, Distribution, Pacific islands, Tonga, Introduction

## Abstract

**Background:**

*Aedes* (*Stegomyia*) *albopictus* is currently one of the most notorious globally invasive mosquito species. Its medical importance is well documented, and its fast expansion throughout most continents is being monitored with concern. It is generally assumed that its expansion through the Western Pacific island countries has not progressed since its establishment in Fiji in 1989. However, the current status of *Ae*. *albopictus* in the Pacific region is largely unknown.

**Findings:**

According to data from the literature and our own observations, *Ae*. *albopictus* is currently present in the following countries of the southern Pacific region: Papua New Guinea, Solomon Islands, Fiji, and the Kingdom of Tonga, where it was first detected in July 2011. It is absent from New Caledonia and French Polynesia where routine entomological surveillance is carried out, and was not detected during entomological work in 2007, either on the Cook Islands or on the Wallis and Futuna Islands. The species was not reported from American Samoa in 2004, but it is mentioned as probably present in Vanuatu. This is the first report of *Ae*. *albopictus* in Tonga.

**Conclusions:**

The introduction and establishment of *Ae*. *albopictus* in Tonga was expected due to the geographical proximity of this country to Fiji where the species is strongly established. The pathway of introduction is unknown. The expansion of *Ae. albopictus* in the Pacific region poses an increasing threat to public health given the role this mosquito plays as primary vector of emerging infectious diseases such as Chikungunya fever.

## Background

The “Asian tiger mosquito” *Aedes (Stegomyia) albopictus* (Skuse, 1895) is nowadays the most notorious invasive Culicidae. From its native range in Asia, the species has spread throughout the Americas, Europe and Africa, mainly via international trade in used tires [1]. Its capacity to transmit a large number of arboviruses is well documented. Although less efficient than *Aedes aegypti* for the dengue viruses transmission, it was responsible for large dengue epidemics in Japan in 1942–1945 [2], for the DEN-1 outbreak in Hawaii in 2001 [3] and for the DEN-2 outbreaks in La Reunion in 1977–78 and 2004 [4,5]. Being the sole vector of the severe chikungunya epidemic in La Reunion and other islands of the Indian Ocean in 2005–06 [6], *Ae. albopictus* was also responsible for a chikungunya outbreak in Italy in 2007 [7] as well as for local chikungunya and dengue transmissions in Southern France in 2010 [8]. *Aedes albopictus* is suspected to play a prominent role in outbreaks of these two diseases in Central Africa [9] and in addition, it is a potential vector of many other arboviruses, including yellow fever, as can be deduced from laboratory experiments [10,11]. Although the species efficiently transmits the dog heartworm *Dirofilaria immitis*[12], it is a poor vector of human filariasis [13].

The distribution of *Ae. albopictus* across the Pacific Island Countries and Territories (PICTs) is patchy. Several islands of the Northern hemisphere like Taiwan and the Japanese islands are part of its native area. However, it is not present on other Northern Pacific islands like Yap in the Federated States of Micronesia, despite continuous traffic of passengers and goods including tires from the neighboring Guam where *Ae. albopictus* is abundant. In Yap, the local *Stegomyia* species *Aedes hensilli* seems to prevent its establishment [14], possibly echoing John Belkin’s 1962 statement: “*Aedes albopictus* does not occur in the South Pacific. It is unlikely that it will become established, for it does not seem to be able to compete with other members of the *scutellaris* group” [15]. Indeed, an intentional introduction of *Ae. albopictus* on a Tuamotu atoll in an attempt to displace the local filariasis vector *Aedes polynesiensis* failed [13]. Eventually, *Ae. albopictus* was reported in Papua New Guinea in 1970[16], in The Solomon Islands in 1979 [17], but only in 1989 in another South Pacific country, namely Fiji [18]. No other introduction has been reported in any other South Pacific island country since then.

A list published in 1995 [19] to which various authors are referring mentions its presence in 11 countries/territories of this region. We assume this is/was not substantiated since (1) the sources are not mentioned, (2) except for the countries previously mentioned, we were unable to find any ancient or recent corroborating data in the literature, and (3) most of the reports do not match our observations.

**Figure 1:**
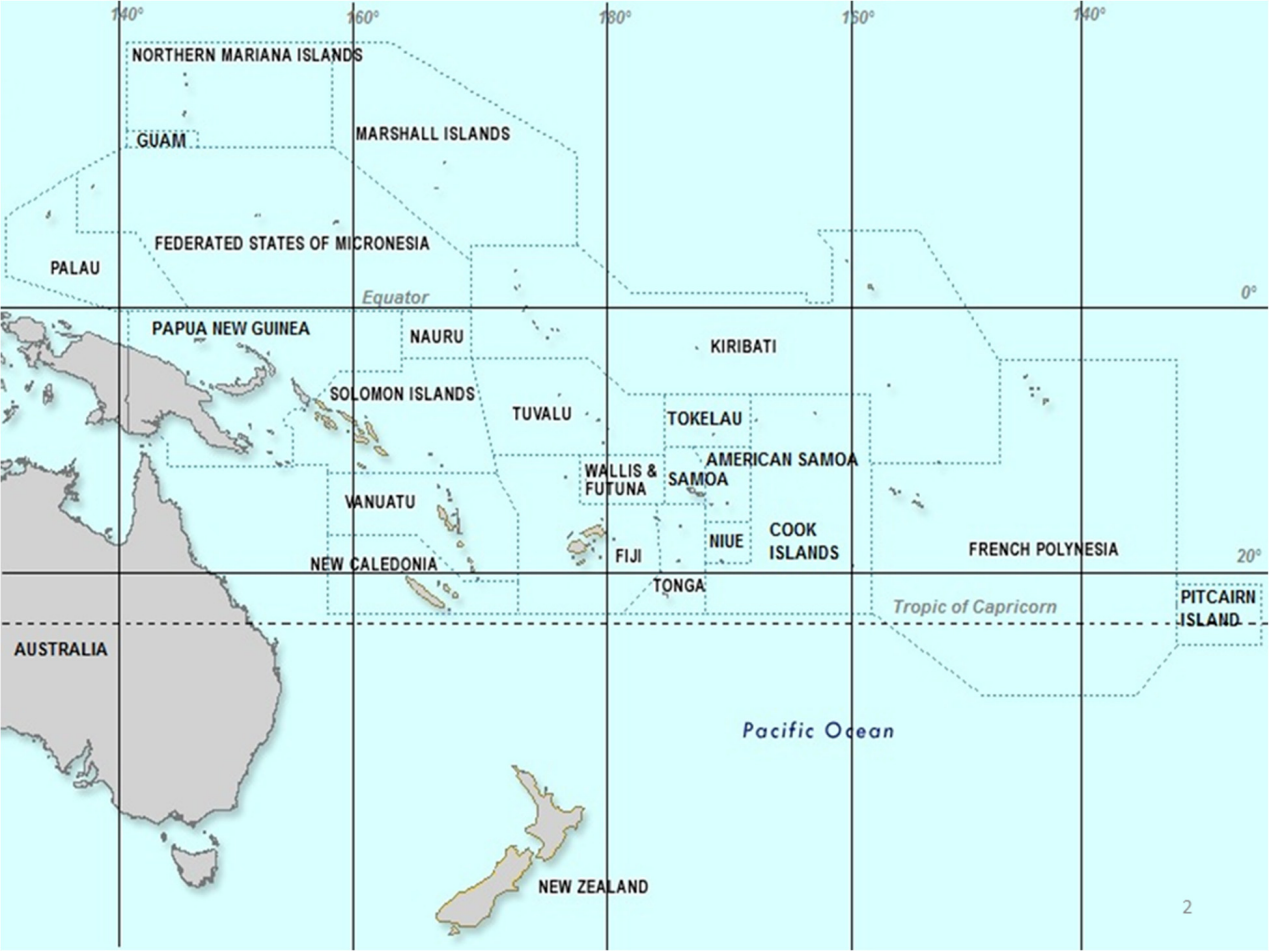
**Map of the South Pacific Ocean. **Modified from WHO with permission.

Here, we describe and discuss the situation with regard to *Ae. albopictus* in all PICTs of the southern hemisphere, and we report the first finding of the species in the Kingdom of Tonga (Figure 1).

## Findings

The overall situation in the PICTs is summarized in Table 1 and more details are given below, except for countries where *Ae. albopictus* is known to have been established for a long time (Fiji, Papua New Guinea, Solomon Islands).

**Table 1:** **Status of *Aedes albopictus *in the 17 South Pacific island countries and territories**

**Countries and Territories**	**Status of *Aedes albopictus***	**References**
Cook Islands	Absent (data from 2007)	IPNC reports
**Fiji**	**Present**	**Laille et al. 1990**
French Polynesia (Tahiti)	Absent	ILM data
Kiribati	Unknown	-
Nauru	Unknown	-
New Caledonia	Absent	IPNC reports
Niue	Unknown	-
**Papua New Guinea**	**Present**	**Cooper et al. 1994**
Pitcairn	Unknown	-
American Samoa	Absent (data from 2004)	Lambdin et al. 2009
Western Samoa	Unkown	-
**Solomon Islands**	**Present**	**Elliott, 1980**
Tokelau	Unknown	-
**Tonga**	**Present**	**This study**
Tuvalu	Unknown	-
Vanuatu	Presence suspected	Chang Moh Seng, pers. com.
Wallis and Futuna	Absent (data from 2007)	IPNC reports

Countries and territories where *Ae*. *albopictus *is present are in bold.

In New Caledonia, the species is **currently absent**. Routine mosquito surveillance, aiming at monitoring dengue and chikungunya vector populations and detecting invasive species around ports of entry (harbor and airport), is carried out using regular larval and adult collections (sticky ovitraps, [20], BG Sentinel® traps, Biogents, Germany) in urban and rural areas. Overall, up to 23,000 mosquitoes are identified annually. *Aedes albopictus* has never been recorded, either on the mainland or on the Loyalty Islands (Institut Pasteur of New Caledonia – IPNC annual reports, unpublished).

In French Polynesia, the species is **currently absent.** Mosquito sampling is frequently conducted for research or surveillance purposes on several archipelagoes (Society Islands, Tuamotu and Marquesas). Sampling is mostly done using ovitraps and BG Sentinel® traps. *Aedes albopictus* has not been detected so far.

On Wallis and Futuna Islands, entomological investigations were carried out by IPNC during a dengue outbreak in 2002 and 2003, and entomological records were updated and extended to the Horne group (Futuna and Alofi islands) in August 2007, supporting that *Ae. albopictus* was **absent**. A total of 3,918 mosquitoes, collected at all stages and in all types of environments including larval sampling in tires and other artificial and natural containers, were identified during these surveys. The prevalent mosquito species was *Ae. polynesiensis*, and a small population of *Ae. aegypti* was found in the main village. No *Ae. albopictus* specimens were found, either on Wallis island or on the Futuna and Alofi islands (IPNC reports, unpublished). However, no field data are available for the last 5 years.

On Cook Islands, the species was **absent in 2007** during the last field investigation on the main island Rarotonga. A training workshop for vector surveillance and control was conducted there in collaboration with the Secretariat of the Pacific Community in September 2007. This entomological training included adult mosquito field trapping (CDC light trap and BG Sentinel® traps) and immature collection targeting container breeding species in both rural and urban settings across the island. The identified dominant species were *Ae. polynesiensis* and *Ae. aegypti*. No *Ae. albopictus* specimen was found. No field data are available for the last 5 years.

A survey conducted in 2004 in American Samoa[21] only reported *Ae. aegypti* and *Ae. polynesiensis*. Thus, it can be assumed that *Ae. albopictus***was absent** from this country up to 2004. The situation is unknown regarding the 7 other countries, namely Kiribati, Nauru, Niue, Pitcairn, Western Samoa, Tokelau and Tuvalu for which, to the best of our knowledge, no studies, unpublished or otherwise or papers have considered the presence of *Ae. albopictus*.

Regarding Vanuatu, no recent specific publications have been found, but according to WHO entomologist (Dr. Chang Moh Seng, personal communication, 2011), the species probably is **currently present** but this needs to be confirmed.

**Figure 2:**
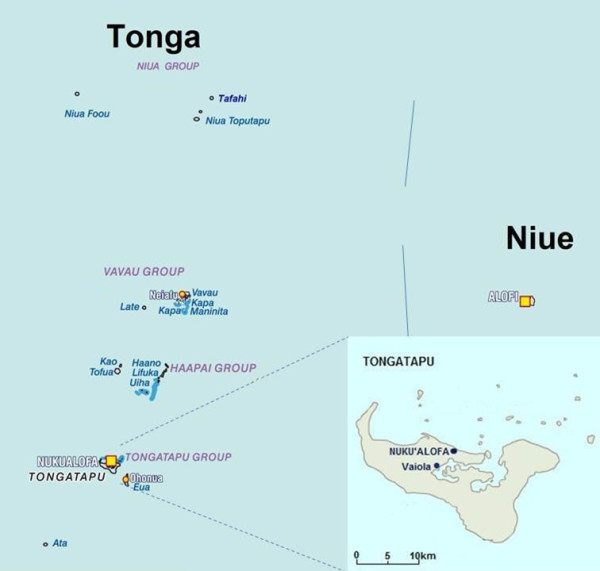
**Map of Tonga showing the localities where *Aedes albopictus *was found. **Modified from WHO with permission.

In Tonga, the species has been **present, at least since 2011.***Aedes albopictus* specimens were collected in June 2011 as part of a training workshop held in Tonga’s capital Nuku’alofa, Immature specimens were found coexisting with *Ae. aegypti* in tires on the waterfront close to the main harbour, and in several types of containers in the locality of Vaiola (Table 2). One adult was captured in a BG Sentinel® trap in Vaiola (Figure 2). Morphological identification was performed using keys and descriptions from Belkin [15]. The risk of misidentification with local *Stegomyia* species was dealt with using complementary documents [22,23]. Some collected individuals were compared to reference specimens from other areas for further confirmation (i.e. Europe, Reunion Island). Finally, molecular confirmation was established by PCR amplification and sequencing of approximately 460 bp of the mitochondrial cytochrome oxidase I (COI) gene [24]. Sequences obtained for 4 specimens (3 females, 1 male) were >99% identical to corresponding *Ae. albopictus* GenBank entries. This is the first report of this species in the Kingdom of Tonga.

**Table 2:** Details of immature mosquito sampling in Tongatapu, 26–30 June 2011

**Locality**	**Sampling place**	**Container type**	***Aedes albopictus***	***Aedes aegypti***
Nuku'Alofa	Waterfront street	Tyre	4	-
Nuku'Alofa	Hotel backyard - Waterfront	Tyre	2	-
Nuku'Alofa	Waterfront street close to cemetery	Tyre	37	14
Vaiola	Auto repair workshop	Large tyre	-	1
Vaiola	Auto repair workshop	4lt metallic can	1	12
Vaiola	Auto repair workshop	Coconut shell	2	-
Vaiola	Auto repair workshop	4lt can with soil	6	-
Vaiola	Auto repair workshop	Small tyre	-	49
Vaiola	Auto repair workshop	Bucket	29	1
Vaiola	Restaurant	Plant pot	6	7
**Total**			**87**	**84**

A prior larval survey conducted in 2007 on the main island of Tongatapu and in the Vava’u group revealed the presence of 8 species including *Ae. aegypti* and the local member of the *Scutellaris* group, *Aedes tongae*[25]. No *Ae. albopictus* was found at the time. *Aedes albopictus* and *Ae. tongae* can be sorted at larval stage owing to clear taxonomic differences such as the saddle, which is complete on the anal segment of *Ae. tongae,* but ventrally interrupted in *Ae. albopictus*[15,26]. This suggests a recent introduction and establishment of *Ae. albopictus* in Tonga.

## Conclusion

Based on literature analyses and our own field investigations, the presence of *Ae. albopictus* is confirmed in 5 out of 17 countries and territories of the Southern Pacific Ocean (Table 1). The establishment in Tonga is not surprising due to its proximity to Fiji where *Ae. albopictus* has been present since 1989, and due to the intense sea and air traffic of passengers and goods between these two countries. Although the classical mode of introduction consists of the transfer of eggs through the tire trade, the invasive mosquito could also have been introduced as larvae or even adults transported in trade ships.

Combined with the emergence of mosquito-borne viruses such as chikungunya in the Pacific region [27], the establishment of *Ae. albopictus* in several PICTs where *Ae*. *aegypti* is already present likely increases the risk of arbovirus transmission since the former species colonizes a broader range of habitats, thus increasing the overall vector density. Consequently, regular vector surveillance to detect the introduction of *Ae*. *albopictus* into new areas should be conducted regularly in PICTs and, if occurring, appropriate vector control measures should be implemented immediately.

While the hypothesis formulated by Belkin to explain the absence of *Ae*. *albopictus* in the south Pacific region might have held true fifty years ago, the situation has dramatically changed with an increase in urbanisation and international trade at levels certainly not predictable at that time. In Fiji and now in Tonga, the presence of local *Stegomyia* species clearly did not prevent *Ae*. *albopictus* from establishing. Undoubtedly, the intensified trade at international and regional level means that many Pacific island countries most likely have encountered *Ae*. *albopictus* on multiple occasions. The reason why *Ae*. *albopictus* has not established in these islands is not clear. The factors driving the success or failure of the establishment of *Ae*. *albopictus* in Pacific island settings are not well understood. The study of *Ae*. *albopictus* on islands where it coexists with local *Stegomyia* species would shed some light on how these species cohabit. The discovery of *Ae*. *albopictus* in new PICTs is of great concern as it may be indicative of a slow but ineluctable trend of invasion through the Pacific region like in other areas of the world.

## Competing interests

The authors declare having no competing interests.

## Authors' contributions

LG carried out the entomological surveillance in New Caledonia, performed the investigations in Wallis and Futuna, Cook Islands and Tonga, and drafted the paper. RO contributed to the field work in Tonga. LS and NS contributed to the field work in Cook Islands and Tonga. HB carried out the entomological surveillance in French Polynesia and edited the manuscript. FS performed the morphological and molecular identification and edited the manuscript. All authors reviewed and approved the final manuscript.
